# Shigellosis seasonality and transmission characteristics in different areas of China: A modelling study

**DOI:** 10.1016/j.idm.2022.05.003

**Published:** 2022-05-23

**Authors:** Zeyu Zhao, Meng Yang, Jinlong Lv, Qingqing Hu, Qiuping Chen, Zhao Lei, Mingzhai Wang, Hao Zhang, Xiongjie Zhai, Benhua Zhao, Yanhua Su, Yong Chen, Xu-Sheng Zhang, Jing-An Cui, Roger Frutos, Tianmu Chen

**Affiliations:** a State Key Laboratory of Molecular Vaccinology and Molecular Diagnostics, School of Public Health, Xiamen University, Xiamen City, People's Republic of China; b CIRAD, UMR 17, Intertryp, Montpellier, France; c Department of Mathematics, School of Science, Beijing University of Civil Engineering and Architecture, Beijing, 102616, People's Republic of China; d Division of Public Health, School of Medicine, University of Utah, 201 Presidents Circle, Salt Lake City, 84112, Utah, USA; e Xiamen Center for Disease Control and Prevention, Xiamen City, Fujian Province, People's Republic of China; f Yichang Center for Disease Control and Prevention, Yichang City, Hubei Province, People's Republic of China; g Longde County Center for Disease Control and Prevention, Guyuan City, Ningxia Hui Autonomous Region, People's Republic of China; h Department of Stomatology, School of Medicine, Xiamen University People's Republic of China; i Public Health England, London, United Kingdom

**Keywords:** Shigellosis, Seasonality, Transmissibility, Early warning, **SEIARW**, Susceptible–Exposed–Infectious/Asymptomatic–Recovered-Water/Food (model), ***R***_***eff***_, effective reproduction number, **MSM**, men who sex with a man, **ARIMA**, Autoregressive Integrated Moving Average (model), **SEIAR**, Susceptible–Exposed–Infectious/Asymptomatic–Recovered (model), **CDC**, Center of Chinese Center for Disease Control and Prevention, **ODE**, ordinary differential equation, **SD**, standard deviation, ***R***_**0**_, basic reproductive number, ***R***^**2**^, Coefficient of determination, **CI**, confidence interval

## Abstract

**Objective:**

In China, the burden of shigellosis is unevenly distributed, notably across various ages and geographical areas. Shigellosis temporal trends appear to be seasonal. We should clarify seasonal warnings and regional transmission patterns.

**Method:**

This study adopted a Logistic model to assess the seasonality and a dynamics model to compare the transmission in different areas. The next-generation matrix was used to calculate the effective reproduction number (*R*_eff_) to quantify the transmissibility.

**Results:**

In China, the rate of shigellosis fell from 35.12 cases per 100,000 people in 2005 to 7.85 cases per 100,000 people in 2017, peaking in June and August. After simulation by the Logistic model, the ‘peak time’ is mainly concentrated from mid-June to mid-July. China's ‘early warning time’ is primarily focused on from April to May. We predict the ‘peak time’ of shigellosis is the 6.30th month and the ‘early warning time’ is 3.87th month in 2021. According to the dynamics model results, the water/food transfer pathway has been mostly blocked off. The transmissibility of different regions varies greatly, such as the mean *R*_eff_ of Longde County (3.76) is higher than Xiamen City (3.15), higher than Chuxiong City (2.52), and higher than Yichang City (1.70).

**Conclusion:**

The ‘early warning time’ for shigellosis in China is from April to May every year, and it may continue to advance in the future, such as the early warning time in 2021 is in mid-March. Furthermore, we should focus on preventing and controlling the person-to-person route of shigellosis and stratified deploy prevention and control measures according to the regional transmission.

## 1 Introduction

Shigellosis, also known as bacterial dysentery, leads to a high incidence in children under five years old in the middle- and low-income countries and is commonly found among travelers and men who have sex with a man (MSM) in the high-income country (Kotloff et al., 2018). Although incidence keeps decreasing in China, the disease burden is unevenly distributed (Zhang et al., 2016, Zhang et al., 2016). For example, the average incidence in Sichuan Province was 22.12 per 100,000 from 2004 to 2014, but the incidence in Hunan Province declined from 15.8 in 2005 to 6.0 per 100,000 in 2010 (Ma et al., 2015; Xu et al., 2017). The peak of incidence and the duration varied widely between Northern and Southern China (Zhang, Ding, et al., 2016).

The seasonality of epidemics of shigellosis is known. Two peaks of shigellosis incidence were reported in Vietnam, with the central peak occurring during the rainy season and a secondary peak in the early or late rainy season (Lee et al., 2017). In China, the peak of shigellosis incidence was from June to August (Zhang, Ding, et al., 2016). The peak in Zhejiang Province occurred between July and October from 2005 to 2010 (Yan et al., 2018). The Logistic model has been used to analyze the relationship between meteorological factors and shigellosis incidence but not to estimate the seasonal epidemic (Zhang, Ding, et al., 2016, Zhang, Si, et al., 2017). However, the Logistic model could evaluate the seasonality of infectious diseases (Liu et al., 2014; Xie et al., 2015, Zhang et al., 2017, Zhang et al., 2017). It is thus possible to build an early warning process for shigellosis based on the Logistic model.

Most studies adopted the Autoregressive Integrated Moving Average model (ARIMA) to predict the trend of shigellosis incidence (Tang et al., 2014; Wang et al., 2016, Zhang, Ding, et al., 2016). However, they did not estimate the transmissibility. The Susceptible–Exposed–Infectious/Asymptomatic–Recovered–Water/Food (SEIARW) model was successfully employed for a shigellosis school epidemic in Changsha City in 2014 (Chen, Leung, et al., 2014). Another study indicated that the SEIARW model could estimate the transmission of shigellosis in Hubei Province and predicted it would be interrupted in 2029 (Chen et al., 2020, Chen et al., 2020, Chen et al., 2020). Then, to estimate transmission for various genders, a sex-based Susceptible–Exposed–Infectious/Asymptomatic–Recovered (SEIAR) model was used (Zhao, Chen, et al., 2020). None of them, however, compared transmission in four locations.

This study calculated the seasonality of reported cases each month in China from 2005 to 2017. We then chose four locations in China to investigate variations in transmissibility.

## 2 Materials and methods

### 2.1 Ethics statement

This disease monitoring and control endeavor was part of the Chinese Center for Disease Control and Prevention's (CDC) normal responsibilities in Yichang City, Xiamen City, Chuxiong City, and Longde County, thus no institutional review or informed consent was necessary. All data analyzed were anonymized.

### 2.2 Data collection

**Fig. 1:**
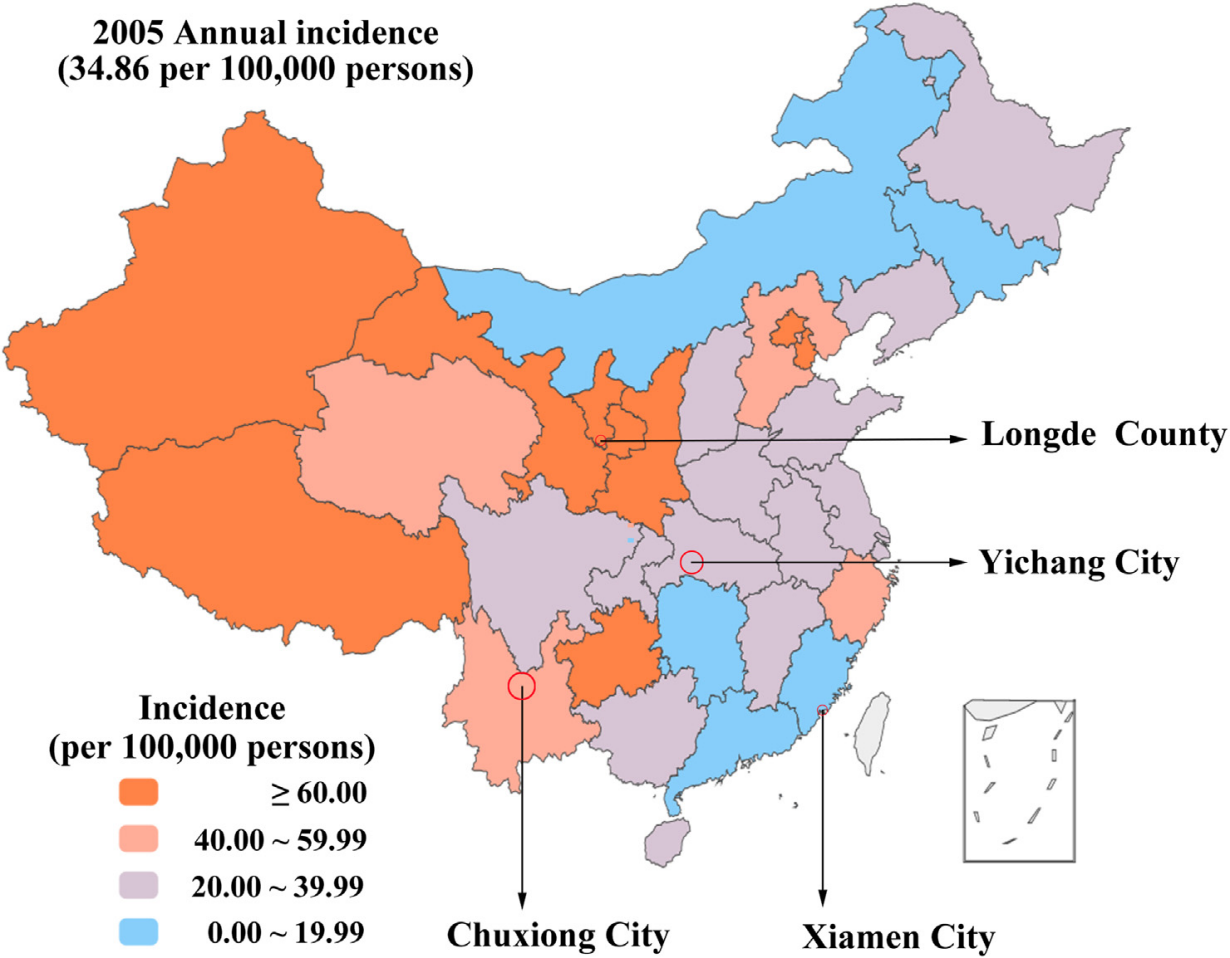
The chosen basis of four areas on the different incidence in 2005 of China.

The provincial dataset of reported shigellosis from all over China (except Hong Kong, Macao, and Taiwan) from 2005 to 2017 was collected from the Public Health Science Data Center of the Chinese Center for Disease Control and Prevention (CDC). It included the monthly reports of cases and incidence of shigellosis in the whole population and separated age groups from 2005 to 2017. The study collected the shigellosis data of each patient from four areas' CDC, i.e., Yichang City from 2005 to 2018, Xiamen City from 2005 to 2018, and Chuxiong Yi Autonomous Prefecture (also known as Chuxiong City) from 2008 to 2018, and Longde County from 2005 to 2018. In the 2005 Shigellosis incidence map, the geographic coordinates of four chosen sites were determined (Fig. 1). The variables collected in the study, including sex, age, and date of onset and the demographic data were collected from the Statistical Yearbook.

### 2.3 Logistic model

The Logistic model is an ordinary differential equation (ODE) model used to describe the self-growth characteristics of biological populations (Tsoularis & Wallace, 2002). The equation is as follows:

(1) $$\frac{dn}{dt}=kn\left(1-\frac{n}{N}\right)$$

The left side of the equation shows the instantaneous rate of cumulative cases (*n*). *k* is the correlation coefficient, and *N* is the upper limit of cumulative cases. After the illness has become saturated in the population, the mean of $1-\frac{n}{N}$ symbolizes the epidemic's growth limit and the creation of the immunological barrier. The onset cases gradually decrease, and the epidemic ends. The Logistic model shows a type of ‘S’ curve, and the disease process leads ‘slow-fast-slow’. The general solution to equation (1) is:

(2) $$n=\frac{N}{1+e^{-kt-c}}$$

*c* represents a constant. According to the characteristics of the Logistic model, it calculates the third derivative of equation (2) and makes it equal to 0, then:

(3) $$\frac{d^{3}n}{dt^{3}}=\frac{Nk^{3}e^{-\left(kt+c\right)}(1-4ke^{-\left(kt+c\right)}+e^{-2\left(kt+c\right)})}{{\left(1+e^{-(kt+c)}\right)}^{4}})=0$$

calculated:

(4) $$t=\frac{-c\pm 1.317}{k}$$

The $t_{1}=\frac{-c-1.317}{k}$ is the turning point of the curve in the model changing from slow to fast (epidemic acceleration). The $t_{2}=-\frac{c}{k}$ is the turning point of the curve changing fastest (the peak of illness onset). The $t_{3}=\frac{-c+1.317}{k}$ is the turning point of the curve in the model, changing from fast to slow.

Any pair of fitted data is substituted into equation (2), which can calculate the parameter *c*, and the month of ‘epidemic acceleration’ per year was calculated by equation (4). We can further calculate the mean and standard deviation (SD) for the month of ‘epidemic acceleration’. Considering that implementation of health decisions and interventions could take a while, we add a SD to the month of ‘epidemic acceleration’, which is defined as ‘early warning time'.

### 2.4 SEIARW model

**Fig. 2:**
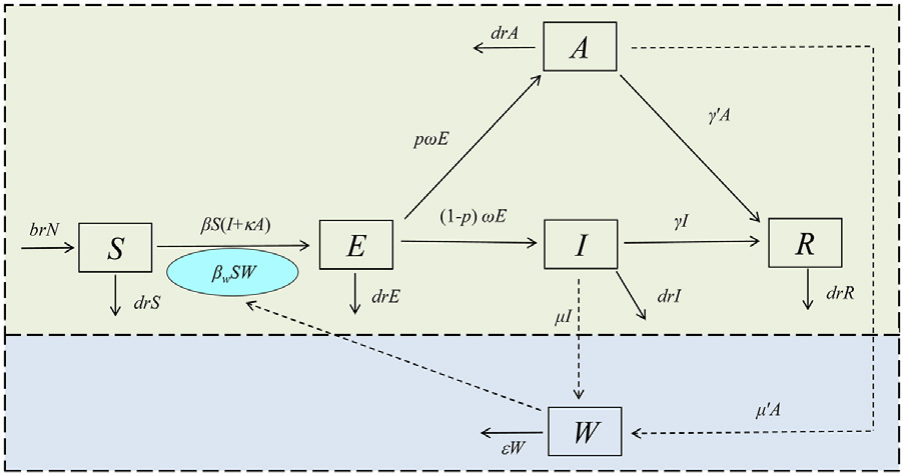
The flowchart of the SEIARW model.

Two transmission routes are considered in this study. The first one is from person-to-person and the second one is water/food-to-person. In the SEIARW model, the compartment *S*, *E*, *I*, *A,* and *R* represent susceptible, exposed, infectious, asymptomatic, and recovered individuals, respectively (Chen et al., 2020, Chen, Leung, et al., 2014; Zhao, Chen, et al., 2020). The compartment *W* refers to water or food in the environment. The definition of the six compartments is provided in Table 1. In the model (Fig. 2), we assume that:

a) The disease does not spread vertically. All persons are equally susceptible. The natural birth rate is *br,* and the mortality rate is *dr.*
b) The susceptible people will be infected after contact with an infected person or contaminated water/food. The transmission rate was *β* and *β*_*w*_, respectively. Meanwhile, we hypothesized that symptomatically infected persons are transmissible at *κ* times the rate of asymptomatically infected people.
c) A proportion (1-*p*) of exposed individuals will change to symptomatically infected people (*I*) following an incubation period. In contrast, the rest (*p*) of exposed individuals will become asymptomatic (*A*) following a latency period (the period during which the exposed individuals become an asymptomatic person).
d) Both symptomatic and asymptomatic infected people will recover at the rate *γ* and *γ*′, respectively, with 1/*γ* and 1/*γ*′ representing their average infectious periods.
e) The infected people shed pathogens into W at rates *μ* and *μ′* respectively for symptomatic and asymptomatic people. We assumed *μ' = cμ.*
f) *Shigella* will die in the water/food after a given time, and the daily decreasing rate of the pathogen is *εW.*

**Table 1:** Definition of six compartments in the SEIARW model.

Variable	Description	Unit
*S*	Susceptible individuals density	Individuals·km^−2^
*E*	Exposed individuals density	Individuals·km^−2^
*I*	Infectious individuals density	Individuals·km^−2^
*A*	Asymptomatic individuals density	Individuals·km^−2^
*R*	Recovered individuals density	Individuals·km^−2^
*W*	Pathogen concentration in water/food reservoir	Cells·mL^−3^
*N*	Total number of population density	Individuals·km^−2^

The differential equation is expressed as follows:

$$\frac{dS}{dt}=brN-\beta S\left(I+\kappa A\right)-\beta_{w}SW-drS$$

$$\frac{dE}{dt}=\beta S\left(I+\kappa A\right)+\beta_{w}SW-\omega E-drE$$

$$\frac{dI}{dt}=\left(1-p\right)\omega E-\gamma I-drI$$

$$\frac{dA}{dt}=p\omega E-\gamma^{\prime }A-drA$$

$$\frac{dR}{dt}=\gamma I+\gamma^{\prime }A-drR$$

(5) $$\frac{dW}{dt}=\mu I+\mu^{\prime }A-\varepsilon W$$

The left side of the equation shows the instantaneous rate of change of *S*, *E*, *I*, *A*, *R,* and *W* at time *t*. In the model adapted the mass action incidence rate [βS(I+κA)] which is better suited to outbreaks with smaller populations (Chen, Leung, et al., 2014). Standard incidence rate [βS(I+κA)/N] is more suitable to simulating epidemics with the large populations according to some studies (Anderson & May 1986; Li et al., 2003). Although we should model based on huge populations using standard incidence rates, we must unify the units between person and pathogen in the reservoir (water/food) (Chen, Leung, et al., 2014; Tien & Earn, 2010). Actually, because the normalized total population can be taken to equal 1, the results produced by normalization are essentially the same as those obtained by using standard incidence rate. The normalized equations are as follows:

*s* = *S/N*, *e* = *E/N*, *i* = *I/N*, *a* = *A/N*, *r* = *R/N*, *w* = *εW/μN*, *b* = *βN*, *b*_*w*_ = *μβ*_*w*_*N/ε*

The equation is as follows:

$$\frac{ds}{dt}=br-bs\left(i+\kappa a\right)-b_{w}sw-drs$$

$$\frac{de}{dt}=bs\left(i+\kappa a\right)+b_{w}sw-\omega e-dre$$

$$\frac{di}{dt}=\left(1-p\right)\omega e-\gamma i-dri$$

$$\frac{da}{dt}=p\omega e-\gamma^{\prime }a-dra$$

$$\frac{dr}{dt}=\gamma i+\gamma^{\prime }a-drr$$

(6) $$\frac{dw}{dt}=\varepsilon \left(i+ca-w\right)$$

### 2.5 Estimation transmissibility

The basic reproductive number (*R*_0_) was always defined as the expected number of secondary infections that result from introducing a single infected individual into an otherwise susceptible population (Chen et al., 2014a, 2019; Zhang et al., 2020). However, we cannot estimate *R*_0_ in this study because we used surveillance data from four regions. Local government intervention measures always influence the wave of surveillance data. We defined the reproduction number estimated after the intervention as an effective reproductive number (*R*_eff_) (Chen et al., 2020, Chen, Leung, et al., 2014). Therefore, we adopt *R*_eff_ to quantify the transmissibility.

In the study, we used next-generation matrix approach to calculate *R*_eff_ (Chen T. M. et al., 2020, Cui et al., 2020). According to equation (5), we can get the disease-free equilibrium point as:

(7) (N,0,0,0,0,0)

$$F=\left[\begin{array}{c} \begin{array}{c} \begin{array}{c} \beta S\left(I+\kappa A\right)+\beta_{w}SW\\ 0 \end{array}\\ 0 \end{array}\\ 0 \end{array}\right],V=\left[\begin{array}{c} \begin{array}{c} \begin{array}{c} \omega E+drE\\ \left(\gamma +dr\right)I+\left(p-1\right)\omega E \end{array}\\ -p\omega E+\left(\gamma^{'}+dr\right)A \end{array}\\ \varepsilon W-\mu I-\mu^{'}A \end{array}\right]$$

(8) $$F=\left[\begin{array}{llll} 0 & \beta S & \beta \kappa S & \beta_{w}S\\ 0 & 0 & 0 & 0\\ 0 & 0 & 0 & 0\\ 0 & 0 & 0 & 0 \end{array}\right],V^{-1}=\left[\begin{array}{llll} \frac{1}{\omega +dr} & 0 & 0 & 0\\ A & \frac{1}{\gamma +dr} & 0 & 0\\ B & 0 & \frac{1}{\gamma^{'}+dr} & 0\\ D & E & F & \frac{1}{\varepsilon } \end{array}\right]$$

In the matrix:

$$A=\frac{\left(1-p\right)\omega }{\left(\omega +dr\right)\left(\gamma +dr\right)}$$

$$B=\frac{p\omega }{\left(\omega +dr\right)\left(\gamma^{\prime }+dr\right)}$$

$$D=\frac{\left(1-p\right)\mu \omega }{\left(\omega +dr\right)\left(\gamma +dr\right)\varepsilon }+\;\frac{\mu^{\prime }p\omega }{\left(\omega +dr\right)\left(\gamma^{\prime }+dr\right)\varepsilon }$$

$$E=\frac{\mu }{\varepsilon \left(\gamma +dr\right)}$$

$$F=\frac{\mu^{\prime }}{\varepsilon \left(\gamma^{\prime }+dr\right)}$$

$$FV^{-1}=\left[\begin{array}{cccc} \beta SA+\beta \kappa SB+\beta_{w}SD & * & * & *\\ 0 & 0 & 0 & 0\\ 0 & 0 & 0 & 0\\ 0 & 0 & 0 & 0 \end{array}\right]$$

$$R_{\text{eff}}=\rho \left(FV^{-1}\right)=\beta S\frac{\left(1-p\right)\omega }{\left(\omega +dr\right)\left(\gamma +dr\right)}+\beta \kappa S\frac{p\omega }{\left(\omega +dr\right)\left(\gamma^{\prime }+dr\right)}+\beta_{w}S\frac{\mu \left(1-p\right)\omega }{\left(\omega +dr\right)\left(\gamma +dr\right)\varepsilon }+\beta_{w}S\frac{\mu^{\prime }p\omega }{\left(\omega +dr\right)\left(\gamma^{\prime }+dr\right)\varepsilon }$$

By the next generation matrix approach, we can get the next generation matrix and *R*_eff_ for the SEAIRW model:

$$R_{\text{eff}}=b\frac{S}{N}\frac{\left(1-p\right)\omega }{\left(\omega +dr\right)\left(\gamma +dr\right)\varepsilon }+\kappa b\frac{S}{N}\frac{p\omega }{\left(\omega +dr\right)\left(\gamma^{\prime }+dr\right)}+b_{w}\frac{S}{N}\frac{\left(1-p\right)\omega }{\left(\omega +dr\right)\left(\gamma +dr\right)}+b_{w}\frac{S}{N}\frac{cp\omega }{\left(\omega +dr\right)\left(\gamma^{\prime }+dr\right)}$$

The ‘knock-out’ simulation, which involves blocking the various shigellosis transmission channels, is another approach for estimating transmissibility (Zhao et al., 2020a, 2020b). In this study, we simulated four ‘knock-out’ situations in this investigation, each shutting off one or two transmission routes: A): *b* = 0; B): *b*_*w*_ = 0; C): *b* and *b*_*w*_ = 0; D): control (no intervention).

### 2.6 Parameter estimation

The definition of parameters *β*, *b*, *β*_*w*_, *b*_*w*_, *κ*, *ω*, *p*, *γ*, *γ*′, *ε*, *c*, *μ*, *μ*′, *br* and *dr* refer to Table 2. The following information or assumptions were used to estimate the parameters:

a) We set parameters *κ*, *γ*′, *ε* and *c* as 0.3125 (range: 0–1), 0.0286 (range: 0–0.0357), 0.6931 (range: 0–1) and 0.3125 (range: 0–1), respectively, based on the epidemiological characteristics of shigellosis and our previous studies (Chen et al., 2020, Chen, Leung, et al., 2014; Zhao, Chen, et al., 2020).
b) The proportions of asymptomatic individuals were reported to range from 0.0037 to 0.27 (Bovee et al., 2012; Khan et al., 2006; Qadri et al., 1995). We set *p* = 0.1 in the SEIARW model.
c) The incubation of shigellosis was reported to range from 1 to 3 days (Debnath et al., 2018; Organization, 2008; Xiao et al., 2012). We assumed the incubation period was equal to the latency period. In the model, we set *ω* as 1 (range: 0.3333–1).
d) The symptoms of shigellosis usually last for a week, but some individuals may experience them for several weeks (Lampel et al., 2018, Prevention, 2020). In the model, we assume that the course of the disease is up to three weeks. We set *γ* to 0.0741 (range: 0.0477–0.1428).
e) The *br* and *dr* were 0.00605 and 0.00524 in Yichang City, 0.01027 and 0.00456 in Xiamen City, 0.0108 and 0.00643 in Chuxiong City, 0.01651 and 0.00758 in Longde County. According to the local Statistical Yearbook, the total population of each year was determined differently in four districts.

**Table 2:** Definition, value and source of all parameters in the SEIARW model.

Parameter	Description	Unit	Value	Range	Method
*β*	Transmission rate from person-to-person	Individuals^−1^·days^−1^	–	≥0	–
*b*	Scaled person-to-person contact rate	Individuals^−1^·days^−1^	–	≥0	Curve fitting
*β*_*w*_	Transmission rate from reservoir-to-person	Individuals^−1^·days^−1^	–	≥0	–
*b*_*w*_	Scaled water/food-to-person contact rate	Individuals^−1^·days^−1^	–	≥0	Curve fitting
*κ*	Relative transmissibility rate of asymptomatic to symptomatic individuals	1	0.3125	0–1	References
*p*	Proportion of the asymptomatic	1	0.1	0.0037–0.27	References
*ω*	Incubation relative rate	days^−1^	1	0.3333–1.000	References
*γ*	Recovery rate of the infectious	days^−1^	0.0741	0.0477–0.1428	References
*γ′*	Recovery rate of the asymptomatic	days^−1^	0.0286	0–0.0357	References
*ε*	Pathogen lifetime rate	days^−1^	0.6931	0–1	References
*c*	Relative shedding rate of asymptomatic compared to infectious	1	0.3125	0–1	References
*μ*	Person–to-reservoir contact rate (“shedding” by Infectious)	cells·mL^−3^ ·day^−1^ ·km^2^ ·individuals^−1^	–	–	–
*μ′*	Person–to-reservoir contact rate (“shedding’’ by Asymptomatic)	cells·mL^−3^ ·day^−1^ ·km^2^ ·individuals^−1^	–	–	–
*br*	Birth rate of the population	1	–	0.00605–0.01651	Statistical Yearbook or CDC
*dr*	Death rate of the population	1	–	0.00456–0.00758	Statistical Yearbook or CDC

-means not applicable.

### 2.7 Simulation method and statistical analysis

Berkeley Madonna 8.3.18 (developed by Robert Macey and George Oster of the University of California at Berkeley; Copyright ©1993–2001 Robert I. Macey & George F. Oster, University of California, Berkeley, CA) was employed for the model simulation. The simulation methods (Runge–Kutta method of order four with tolerance set to 0.001) were the same as those previously published research (Chen et al., 2016, Chen et al., 2020; Liu et al., 2015). Berkeley Madonna adopted the curve fitting of the least root-mean-square deviation. GraphPad Prism 7.0 (GraphPad Software, La Jolla, CA) and Microsoft Office Excel 2019 (Microsoft, Redmond, WA, USA) were employed for the figure development and data analysis. All the maps presented in this study were created with Pyecharts 1.2.1 (2017–2019. Powered by docsify), a package from Python software, version 3.6.1 (Copyright 2001–2017; Python Software Foundation, Powered by Heroku). The coefficient of determination (*R*^2^) calculated SPSS 21.0 (IBM Corp, Armonk, NY, USA) was adopted to judge the model goodness of fit. The Linear model in SPSS 21.0 was used to fit and predict the data of peak and early warning time.

### 2.8 Sensitivity analysis

In our model, the seven parameters were split into 1,000 values according to their range. The value of mean and mean ± SD was exited after the sensitivity analysis in the model. As the simulation method was the same in each year with different areas, the sensitivity analysis was just performed for 2009 data of Longde County.

## 3 Results

### 3.1 Epidemiological characteristics of shigellosis

**Fig. 3:**
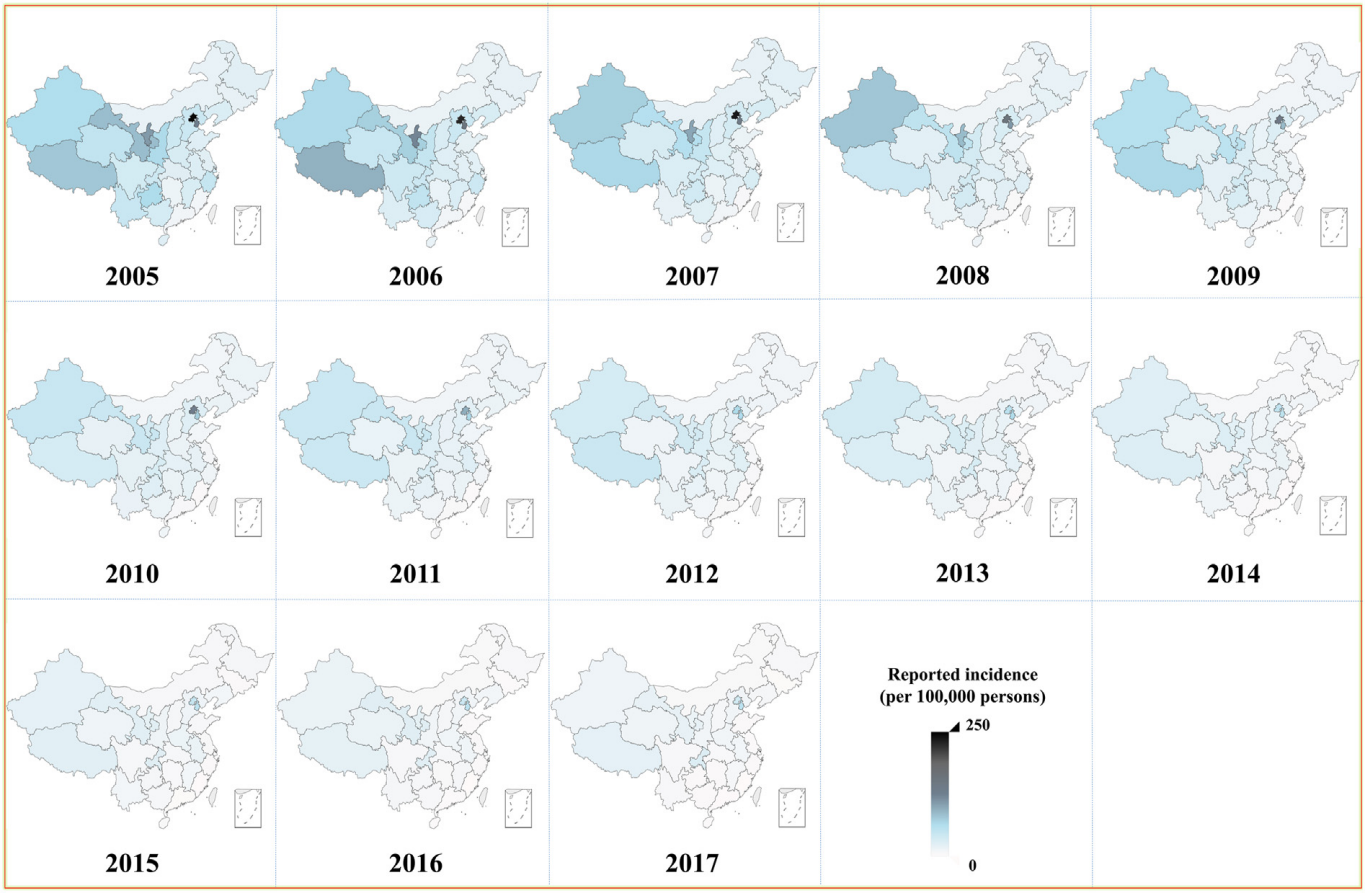
The reported incidence of shigellosis from 2005 to 2017 in China (excepted Hong Kong City, Macao City, and Taiwan Province).

In China (excluding Hong Kong, Macao, and Taiwan), the incidence of shigellosis has fallen from 35.12 cases per 100,000 in 2005 to 7.85 cases per 100,000 in 2017 (Fig. 3). The reported incidence varied in different areas. The top medians of reported incidence were 94.35/100,000 in Beijing City, 65.50/100,000 in Tianjin City, 42.87/100,000 in Gansu Province, 41.08/100,000 in Xinjiang Uygur Autonomous Region, 40.56/100,000 in Tibet Autonomous Region, and 33.69/100,000 in Ningxia Hui Autonomous Region.

**Fig. 4:**
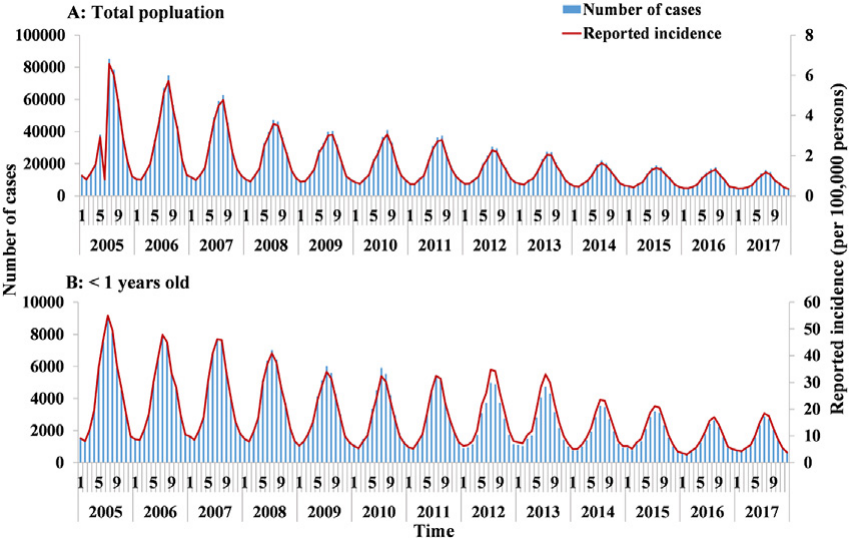
The number of cases and reported incidence per month of shigellosis from 2005 to 2017 in total population and < 1 years old. (A) Total population; (B) < 1 years old.

**Fig. 5:**
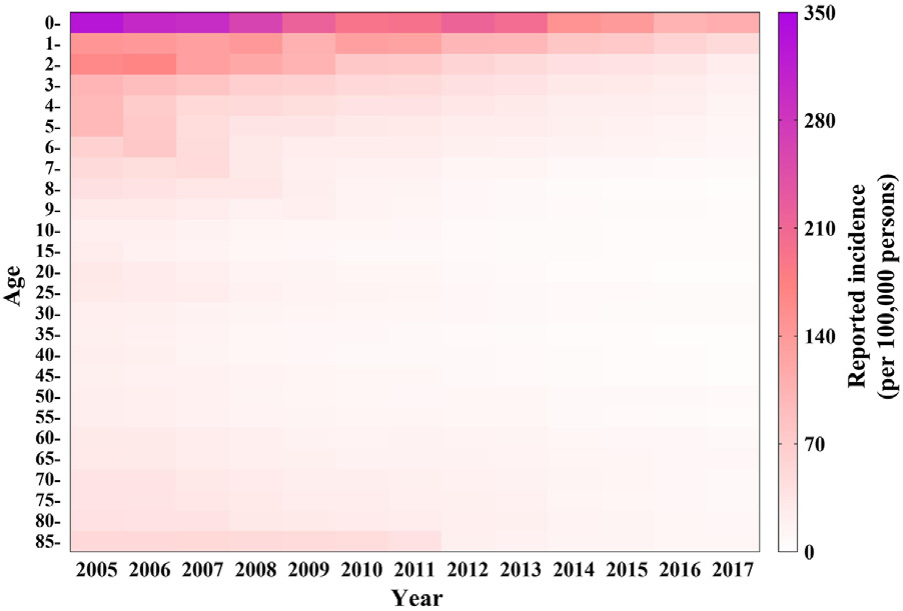
The reported incidence of shigellosis from 2005 to 2017 in different age groups.

The reported cases and incidence per month seasonally decreased from 2005 to 2017, in the whole population and among infants under 1 (Fig. 4). The reported incidence in the total population (median: 1.05/100,000) showed a decreasing trend. Individuals < 5 years old displayed a high incidence, especially those under 1 (median: 13.01/100,000), from 2005 to 2017 (Fig. 5).

**Fig. 6:**
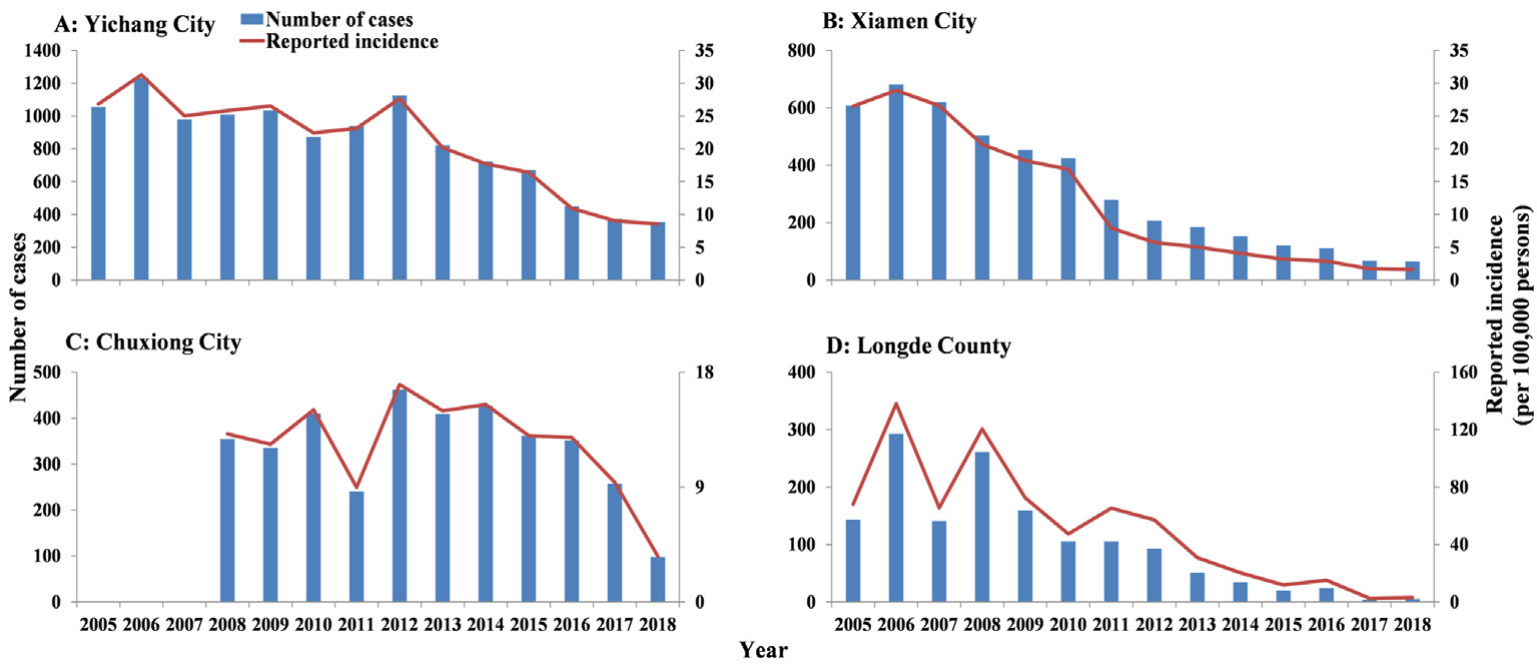
The number of cases and the reported incidence of shigellosis in Yichang City, Xiamen City, Chuxiong City, and Longde County. (A) Yichang City; (B) Xiamen City; (C) Chuxiong City; (D) Longde County.

In Fig. 6, the reported incidence displayed a decreasing trend in Yichang City, Xiamen City, and Long County. However, it showed an increasing trend before 2012 in Chuxiong City. The medians of reported incidence were 52.20/100,000, 22.78/100,000, 13.03/100,000, and 6.83/100,000 in Longde County, Yichang City, Chuxiong City, and Xiamen City, respectively.

**Fig. 7:**
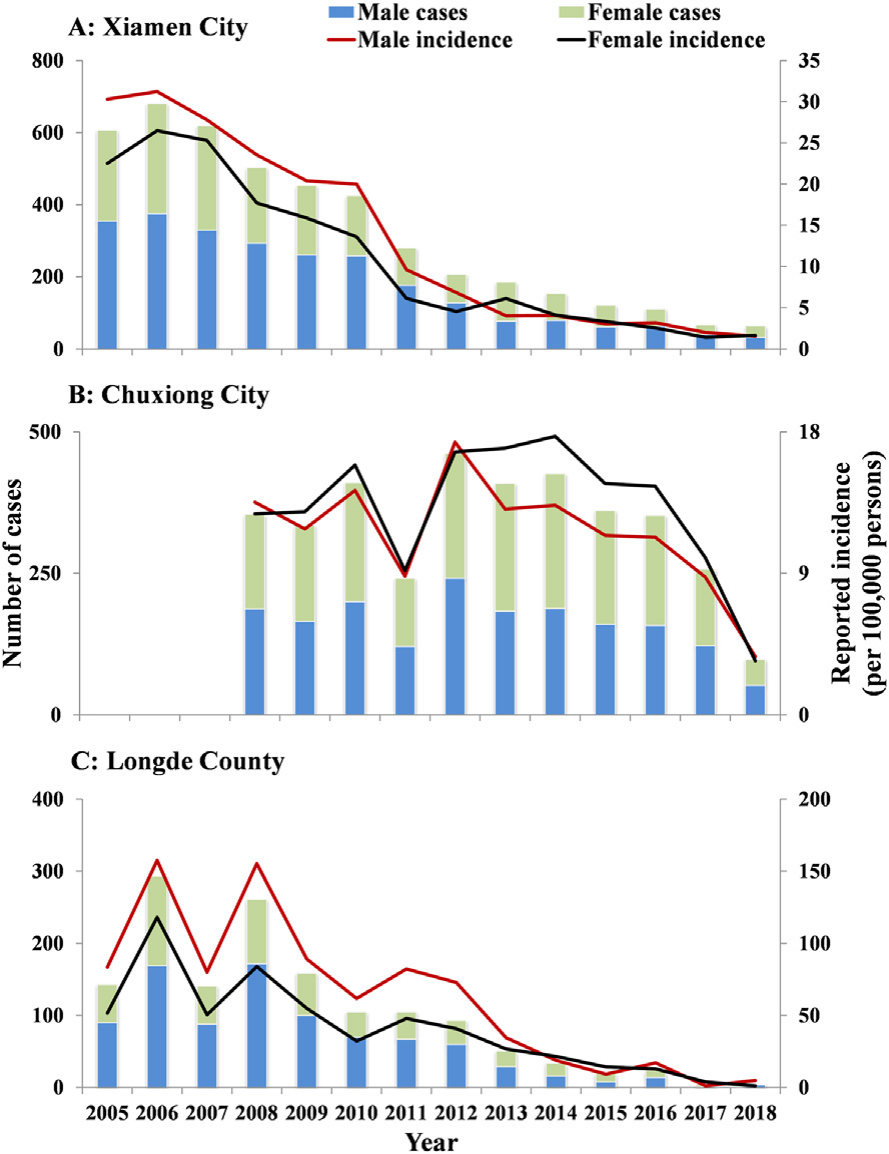
The number of cases and reported incidence between males and females in Xiamen City, Chuxiong City, and Longde County. (A) Xiamen City; (B) Chuxiong City; (C) Longde County.

**Fig. 8:**
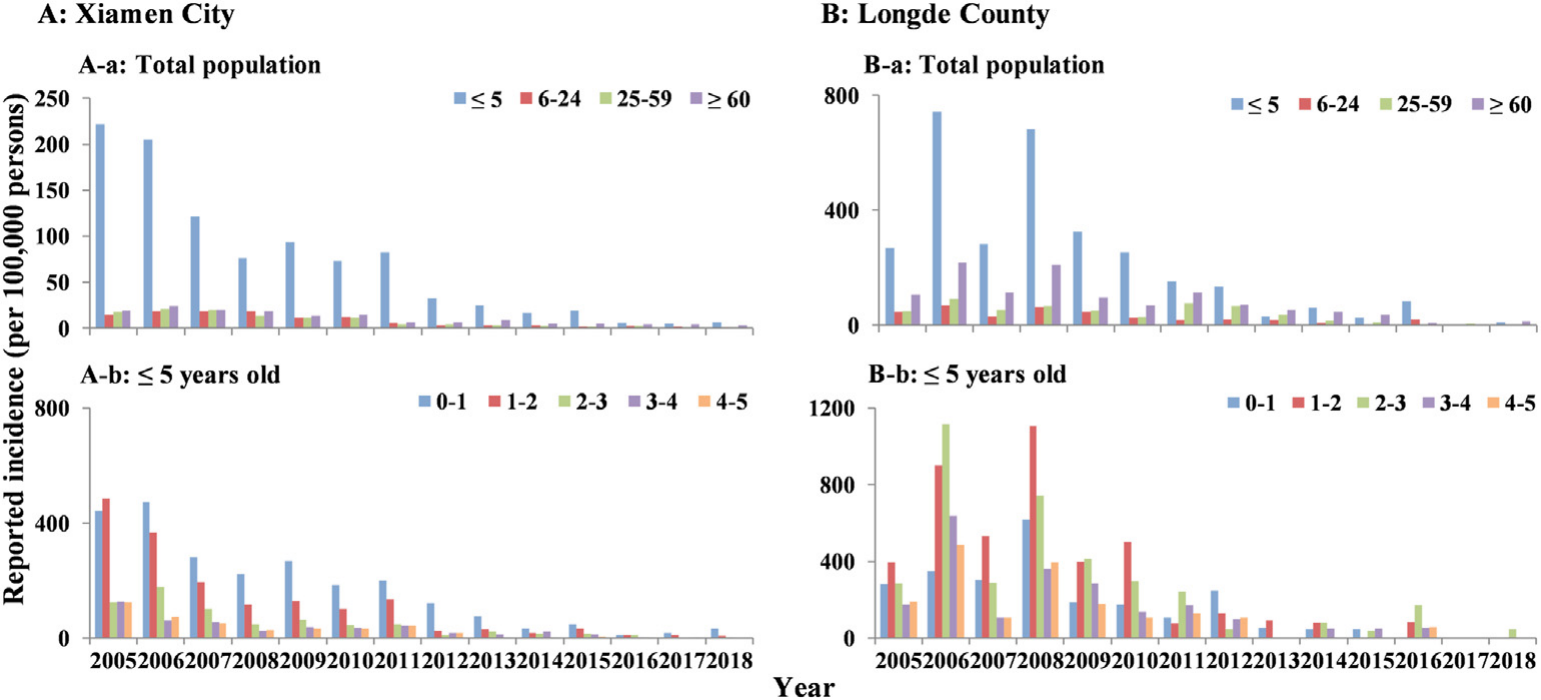
The reported incidence of shigellosis from 2005 to 2018 in different age groups in Xiamen City and Longde County. (A) Xiamen City: (A-a) total population, (A-b) ≤ 5 years old; (B) Longde County: (B-a) total population, (B-b) ≤ 5 years old.

The median incidence in males was higher than in females in Xiamen City (males: 8.22/100,000, females: 6.11/100,000) and Longde County (males: 67.36/100,000, females: 36.58/100,000). However, in Chuxiong City, the median incidence was higher in females than males (males: 11.82/100,000, females: 14.54/100,000) (Fig. 7). There was an increased incidence in children aged ≤ 5 years old (median: 53.09/100,000) in Xiamen City, especially in children under 2. An increased incidence was reported in Longde County in children aged ≤ 5 years old (median: 143.48/100,000), especially in ≤ 3 years children and elder aged ≥ 60 years old (median: 70.49/100,000) (Fig. 8).

### 3.2 Curve fitting results

**Fig. 9:**
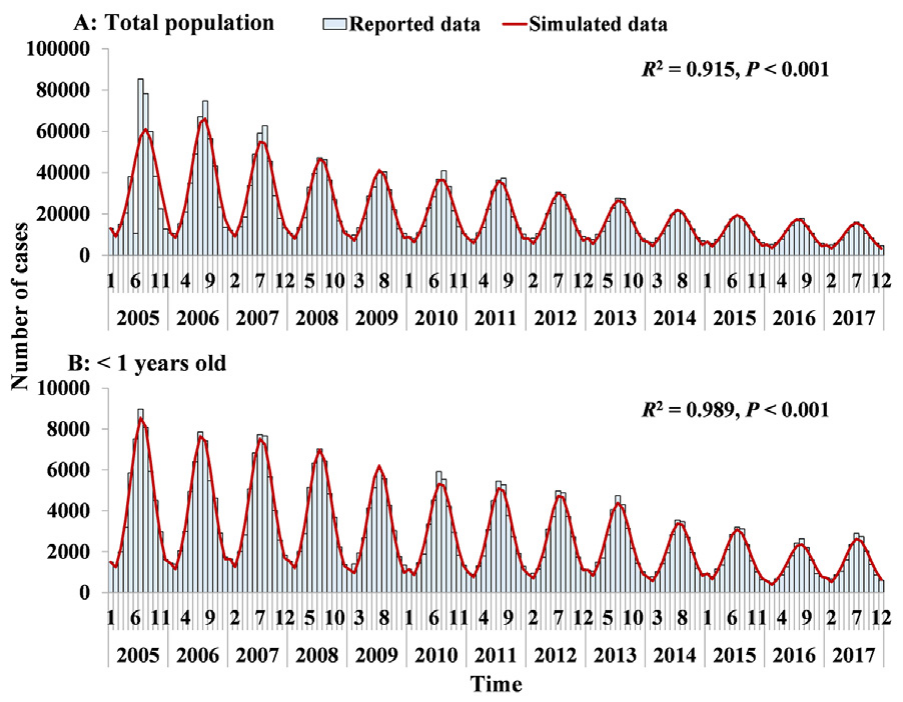
Curve fitting of Logistic model to the reported data per month from 2005 to 2017. (A) Total population; (B) < 1 years old.

The Logistic model (Fig. 9) had a very positive fitting effect on the reported data (Total population: *R*^2^ = 0.915, *P* < 0.001; < 1 years old: *R*^2^ = 0.989, *P* < 0.001).

**Fig. 10:**
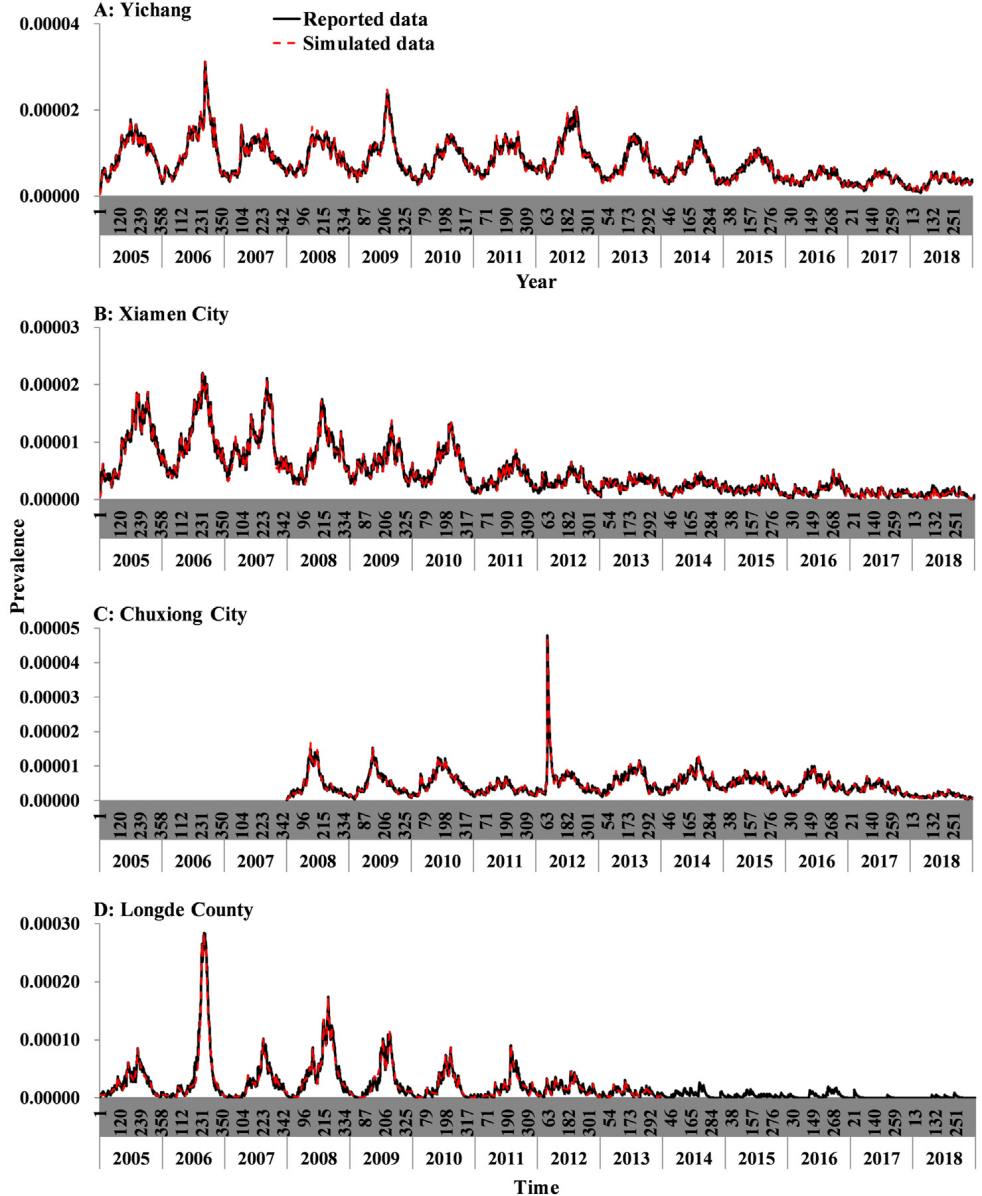
Curve fitting of SEIARW model to the reported data per day in four areas. (A) Yichang City; (B) Xiamen City; (C) Chuxiong City; (D) Longde County.

The SEIARW model (Fig. 10) fitted the data effectively (Yichang City: *χ*^2^ = 0.0003, *P* > 0.999; Xiamen City: *χ*^2^ = 0.0007, *P* > 0.999; Chuxiong City: *χ*^2^ = 0.0005, *P* > 0.999; Long County: *χ*^2^ = 0.0087, *P* > 0.999). Owing to the limited number of reported cases after 2013 in Longde City, we just modeled the data from 2005 to 2013.

### 3.3 Seasonal characteristics of shigellosis

**Fig. 11:**
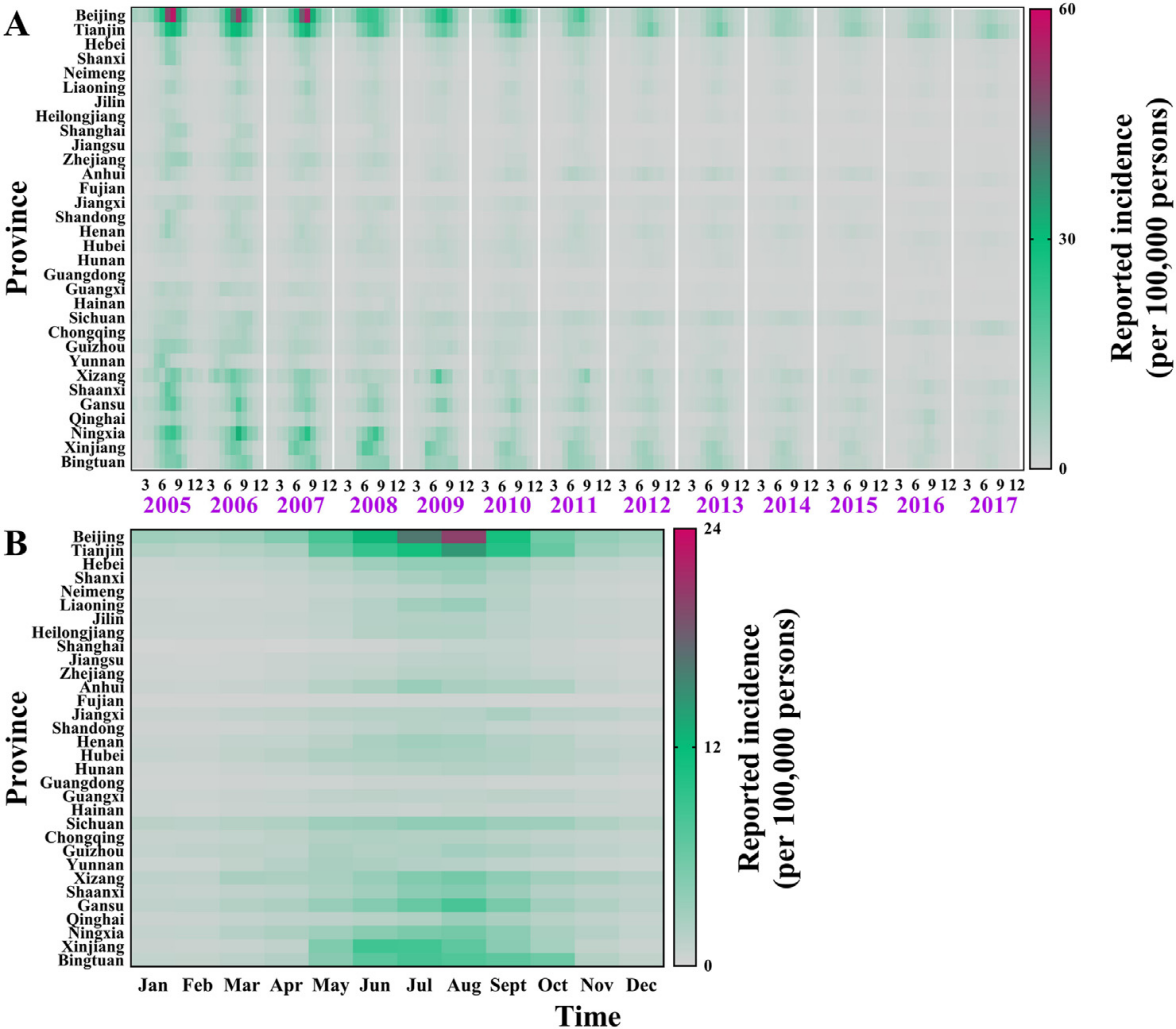
Reported incidence and median incidence per month in each province of China from 2005 to 2017. (A) Reported incidence per month from 2005 to 2017; (B) Median monthly incidence from 2005 to 2017.

**Fig. 12:**
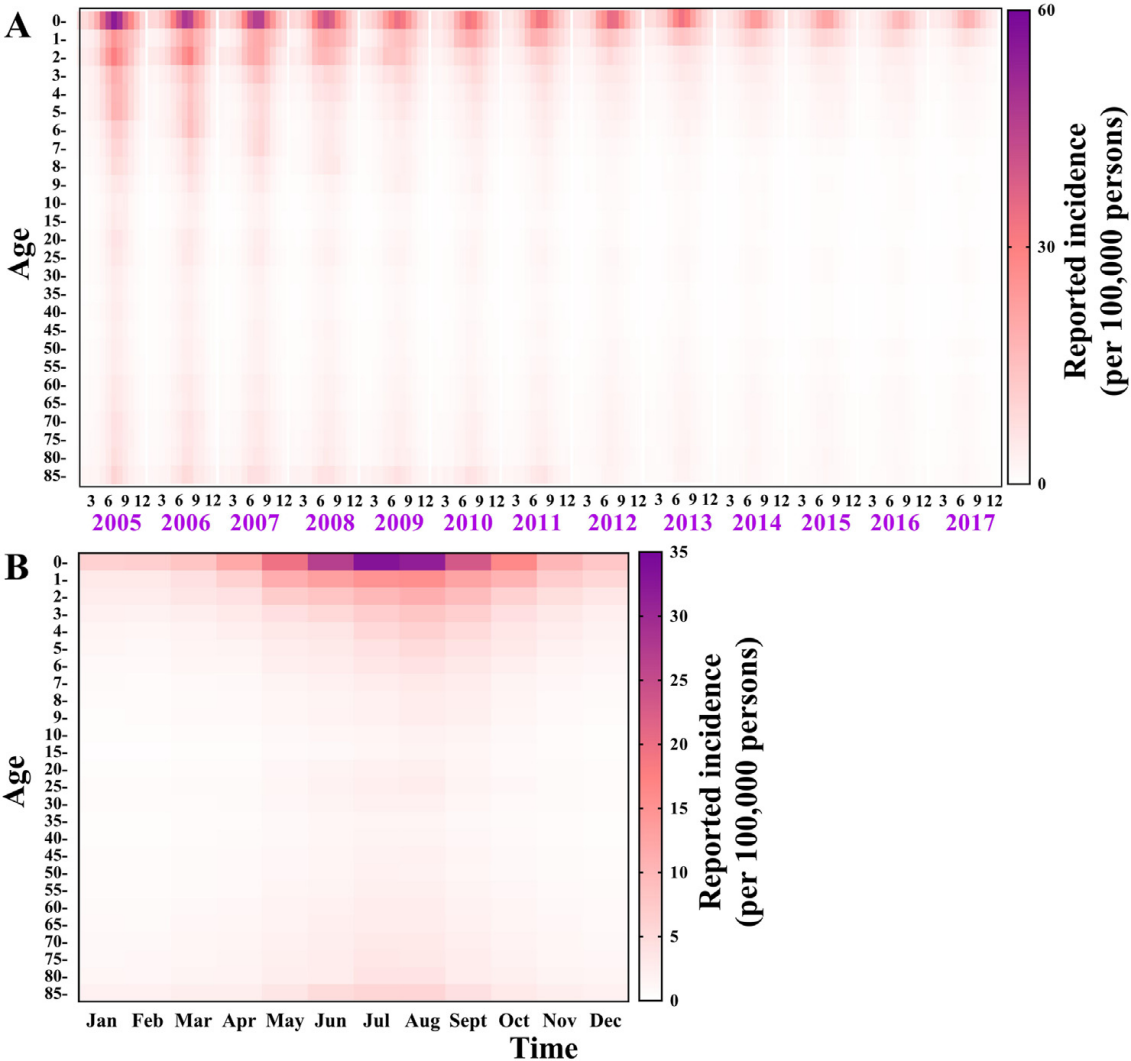
Reported incidence and median incidence per month in each age group of China from 2005 to 2017. (A) Reported incidence per month from 2005 to 2017; (B) Median monthly incidence from 2005 to 2017.

There is a seasonal transmission characteristic of shigellosis in all provinces in China (Fig. 11), which is most common from May to October (Fig. 11B). The incidence for each age group also shows a seasonality. The incidence of each age group slowly increases from January to May; the peak is from June to August, then gradually decreases from September to December (Fig. 12B).

**Fig. 13:**
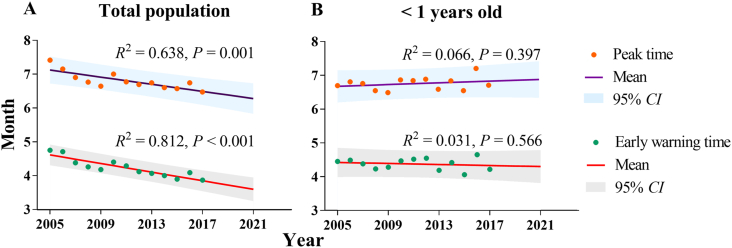
Fitting and prediction of peak and early warning time by Linear model. (A) Total population; (B) < 1 years old.

From 2005 to 2017, the result of the Logistic model in the total population (Table 3) located the mean of ‘epidemic acceleration’ at 4.50 months (SD: 0.27) and of “peak incidence” corresponded to is 6.76 months (range: 5.49–6.43 months). Meanwhile, the ‘epidemic acceleration month’ was gradually shortened year after year. Thus, the ‘warning time’ was 4.23 months in the total population. In children < 1 years old (Table 4), the mean month of ‘epidemic acceleration’ was 4.55 months (SD: 0.17), and the ‘peak incidence’ was at 6.78 months (range: 5.51–6.23). Therefore, the ‘early warning time’ should be 4.38 months. The results of Linear model (Fig. 13) show the decreasing trend of ‘peak time’ (*R*^2^ = 0.638, *P* = 0.001) and ‘early warning time’ (*R*^2^ = 0.812, *P* < 0.001) in total population. We predict the ‘peak time’ is 6.30 months and ‘early warning time’ is 3.87 months in the total population of 2021. There is slowly rising trend in the ‘peak time’ (*R*^2^ = 0.066, *P* = 0.397) and slowly decreasing trend in the ‘early warning time’ (*R*^2^ = 0.031, *P* = 0.566) in < 1 years old. We predict the ‘peak time’ is 6.90 months and ‘early warning time’ is 4.47 months in < 1 years old of 2021.

**Table 3:** Simulation results of Logistic model in total population.

Year	*k*	*N*	*c*	*t*_1_	Peak/*t*_2_
2005	0.5468	0.00035	−4.06	5.02	7.43
2006	0.5999	0.00034	−4.30	4.98	7.17
2007	0.5784	0.00030	−4.00	4.65	6.92
2008	0.5842	0.00024	−3.96	4.53	6.78
2009	0.5951	0.00021	−3.96	4.45	6.66
2010	0.5597	0.00020	−3.93	4.67	7.02
2011	0.5889	0.00018	−4.00	4.56	6.79
2012	0.5675	0.00016	−3.81	4.39	6.71
2013	0.5440	0.00015	−3.68	4.34	6.76
2014	0.5599	0.00012	−3.71	4.27	6.62
2015	0.5444	0.00011	−3.59	4.17	6.59
2016	0.5494	0.00009	−3.71	4.36	6.76
2017	0.5610	0.00008	−3.64	4.14	6.49

**Table 4:** Simulation results of Logistic model in under 1 years old.

Year	*k*	*N*	*c*	*t*_1_	Peak/*t*_2_
2005	0.6264	0.00339	−4.21	4.62	6.72
2006	0.6082	0.00311	−4.15	4.66	6.83
2007	0.5910	0.00309	−4.01	4.55	6.78
2008	0.6072	0.00270	−3.99	4.40	6.57
2009	0.6391	0.00221	−4.16	4.45	6.51
2010	0.5862	0.00203	−4.04	4.64	6.89
2011	0.6048	0.00205	−4.16	4.69	6.87
2012	0.6017	0.00225	−4.16	4.72	6.91
2013	0.5840	0.00211	−3.86	4.36	6.61
2014	0.5819	0.00158	−3.99	4.59	6.86
2015	0.5609	0.00147	−3.69	4.23	6.57
2016	0.5466	0.00113	−3.95	4.82	7.23
2017	0.5639	0.00120	−3.79	4.39	6.73

### 3.4 Transmissibility of shigellosis in different areas

**Fig. 14:**
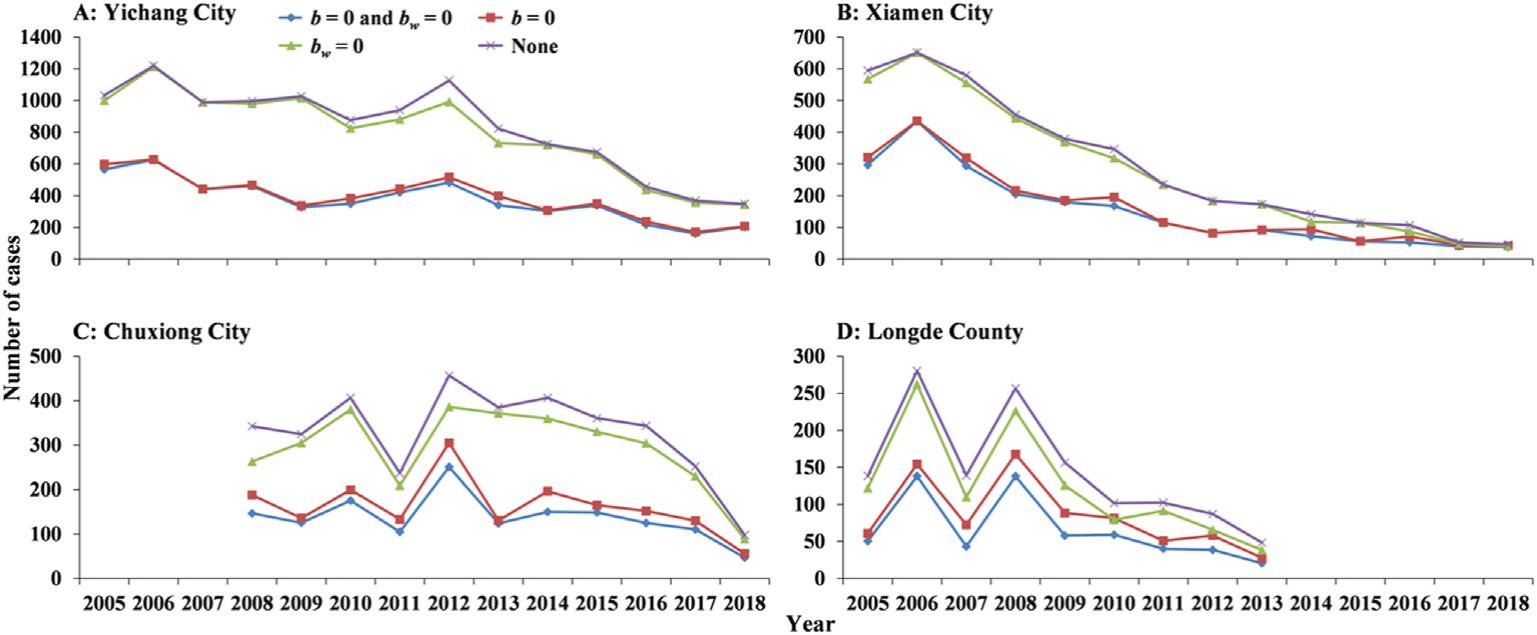
‘Knock-out’ simulation in Yichang City, Xiamen City, Chuxiong City, and Longde County. (A) Yichang City; (B) Xiamen City; (C) Chuxiong City; (D) Longde County.

In Yichang City and Xiamen City, the ‘knock-out’ simulation (Fig. 14) showed that the number of cases in scenario B (*b*_*w*_ = 0) was consistent with that in scenario D (control). Meanwhile, the number of cases in scenario A (*b* = 0) was consistent with scenario C (*b* and *b*_*w*_ = 0). However, in Chuxiong City and Longde County, the number of cases in scenario B (*b*_*w*_ = 0) was slightly different from scenario D (control).

**Fig. 15:**
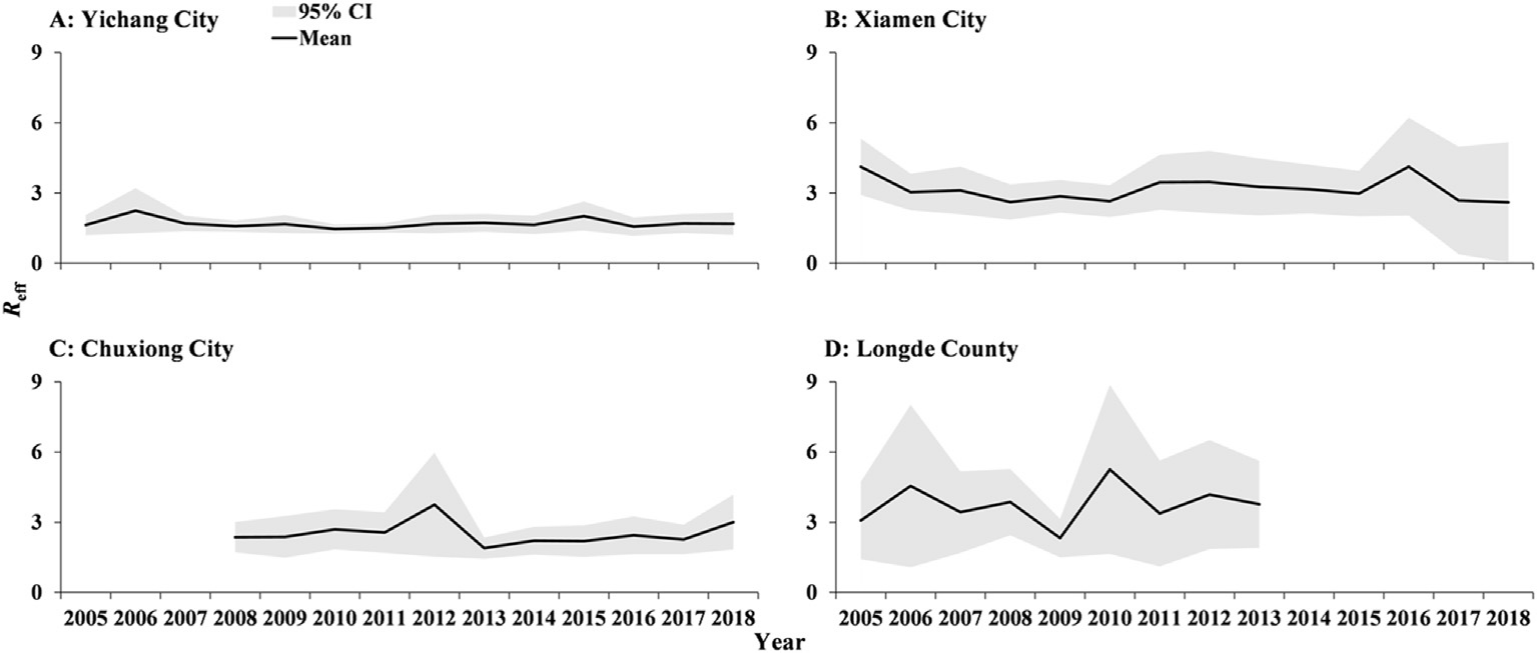
The transmissibility (*R*_*eff*_) comparison among Yichang City, Xiamen City, Chuxiong City, and Longde County. (A) Yichang City; (B) Xiamen City; (C) Chuxiong City; (D) Longde County.

**Fig. 16:**
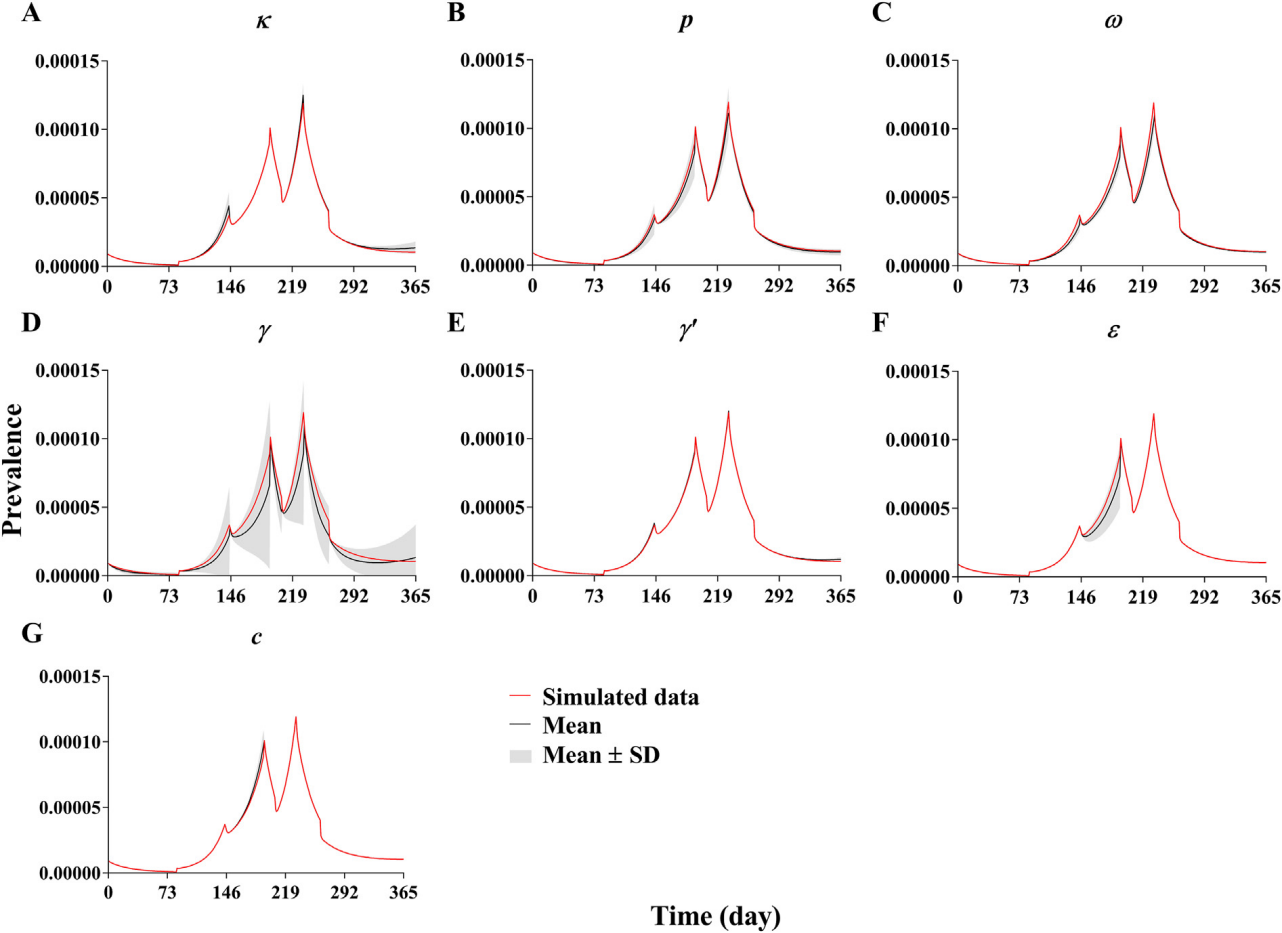
Sensitivity analysis of SEIARW model in Longde County of 2009. (A) *κ* = 0.3125 (0–1); (B) *p* = 0.1 (0.0037–0.27); (C) *ω* = 1 (0.3333–1); (D) *γ* = 0.0741 (0.0477–0.1428); (E) *γ*’ = 0.0286 (0–1); (F) *ε* = 0.6931 (0–1); (G) *c* = 0.3125 (0–1).

The results of *R*_eff_ showed a higher order of transmissibility (Fig. 15) in Longde County (Mean: 3.76, 95% confidence interval [CI]: 1.28–2.11) than in Xiamen City (Mean: 3.15, 95% CI: 1.87–4.43), Chuxiong City (Mean: 2.52, 95% CI: 1.63–3.41) and Yichang City (Mean: 1.70, 95% CI: 1.28–2.11). SEIARW model was not sensitive to parameters *γ*’ and *c*. The model had moderate sensitivity for parameters *κ*, *p*, *ω*, and *ε*, and a lot of sensitivity for parameter *γ* (Fig. 16).

## 4 Discussion

### 4.1 Model validity

The Logistic model estimated seasonal influenza and established the early warning mechanism (Cheng et al., 2018; Míguez et al., 2016). According to *R*^2^ of the linear regression, the Logistic model exhibited high goodness of fit with the reported data. This finding suggests that the model was suitable for estimating the seasonal characteristics of shigellosis. The method of the Logistic model could also be used to estimate the different epidemic stages.

The chi-square results showed that the SEIARW model was suitable for estimating shigellosis transmission, which was consistent with the results of previous research (Chen et al., 2020, Chen, Leung, et al., 2014). The model was more sensitive to parameter *γ*. This finding suggests that we should collect the parameter from actual data instead of the literature.

### 4.2 Epidemiological characteristics

From 2005 to 2017, the descriptive data revealed a declining trend in shigellosis. Beijing City, Tianjin City, Gansu Province, Xinjiang Uygur Autonomous Region, Tibet Autonomous Region, and Ningxia Hui Autonomous Region had the most significant occurrences. Except in Beijing and Tianjin, this incidence feature is directly tied to economic situations. Shigellosis has a lower incidence in areas with better economic conditions (von Seidlein et al., 2006). However, the high incidence of shigellosis in Beijing City and Tianjin City may be related to a high population density and low traveling population. Among the four selected areas, the incidence in Longde City is higher than in Yichang City, which was higher than in Chuxiong City and higher than in Xiamen City. The economic conditions in Longde County and Chuxiong City areas are much lower than those found in Xiamen City and Yichang City. However, the frequency of flood disasters in Yichang City is higher than in other areas, which leads to a relatively higher incidence. For example, the rainfall in Yichang City mainly increased and there were 12 times of rainstorms in 2007, and the rainstorm caused heavy disasters in 6 counties of Yichang City in 2020 (Organization, 2017).

Most studies have indicated that shigellosis remains a high disease burden in children ≤ 4 years old (Kotloff et al., 2013, 2018; Liu et al., 2016). This is consistent with our results showing that the disease occurs mainly in children under five years old. Meanwhile, the results showed that the various areas had reported different incidences between genders and several age groups.

### 4.3 Seasonal characteristics of shigellosis

In this study, the Logistic model showed that the peak of incidence was at 6.76 months (range: 5.49–6.43 months), and the ‘early warning time’ was 4.23 months. Although the shigellosis incidence is decreasing in China, it is essential to build an early warning process, especially for children under five (Kotloff et al., 2018). Our results suggest that we might implement the prevention and control measures in early April every year. Meanwhile, in summer and autumn, the factors such as reduced air pressure, abundant rainfall, and easy breeding of bacteria, provide suitable conditions for the transmission of *Shigella* (Das et al., 2018; Farag et al., 2013; Xu et al., 2014). A shigellosis research in Chongqing City found that the incidence was highest from May to October (Meng et al., 2019), while another study in Zhejiang Province found that the incidence was highest in the summer and fall from July to October (Yan et al., 2018). Indeed, according to our description, there was heterogeneity of seasonality in different provinces. We assume that it is related to the difference in meteorological, economic, and demographic factors. There exist differences in rainfall, sunshine hours, and temperature between southern and northern China. The economic conditions in the south are better than those in the north, and the former has a relatively high population density. In addition, we observed the seasonal heterogeneity in different age groups, suggesting that we should re-build the early warning mechanism for the age group with a heavy disease burden. Furthermore, we found a decreasing trend of ‘peak time’ and ‘early warning time’ in the total population, but a stable trend in < 1 years old, which may relate to the decrease in shigellosis incidence or the reproduction of *shigella*. Meanwhile, this finding suggests that we should establish the early warning mechanism at the end of March to control the transmission in the total population and in the early of April to prevent the transmission in < 1 years old.

### 4.4 Transmissibility of shigellosis in different areas

The ‘knock-out’ simulation revealed that water and food have a little role in the transmission of shigellosis, suggesting that the disease is predominantly transmitted from person to person. This is congruent with the results of other studies (Chen et al., 2020, Kotloff et al., 2018; Zhao, Chen, et al., 2020). However, it contradicts another study that concluded that water and food had a high contribution to shigellosis transmission in a school outbreak (Chen, Leung, et al., 2014). Furthermore, our model was constructed on the complete population and has been applied to surveillance datasets for many years. It is very different from shigellosis transmission features in a specific outbreak.

Our work further indicated that the transmissibility in Longde County was higher than in Xiamen City, followed by Chuxiong City and Yichang City, which might be due to the lower economic situation in Longde County and Chuxiong City than in Yichang City and Xiamen City. According to the Statistical Yearbook, Xiamen City, a Chinese special economic zone, had 4,110,000 population in 2018 over a surface of 1,700.61 km^2^. However, Yichang City reported 4,135,900 residents for 21,084 km^2^. The contact frequency and the travel population in Xiamen City should be much higher than Yichang City. Our finding suggests that decreasing the travel population and contact frequency might reduce the transmission of shigellosis.

## 5 Limitations

Because this study, like many modeling studies, relies on assumptions, the truth may be more complex. The logistic model was used at the national level, but more specific evaluations of individual provinces or cities are required. Another restriction is that, while we studied transmissibility in different places, aspects such as weather, economics, and demography were not included in our datasets and could not be analyzed in this study. We need to explore further the economic, environmental, and cultural influences which could correlate with shigellosis transmission.

## 6 Conclusions

Shigellosis remains a high disease burden in China, especially for children under 1. The month of the ‘peak time’ is 6.76 months, ‘epidemic acceleration’ is 4.50 months, and the recommended ‘early warning time’ is 4.23 months. The seasonality and transmission features are different in several areas. The prevention and control measures should be applied, especially in low-income provinces in China.

## Ethics approval and consent to participate

Institutional review and informed consent were not required for this study. All data analyzed were anonymized.

## Consent for publication

Not applicable.

## Availability of data and materials

Data supporting the conclusions of this article are included within the article.

## Funding

This study was partly supported by the Bill & Melinda Gates Foundation (INV-005834), the Science and Technology Program of Fujian Province (No: 2020Y0002), and the Xiamen New Coronavirus Prevention and Control Emergency Tackling Special Topic Program (No: 3502Z2020YJ03).

## Authors' contributions

Conceptualisation: Tian-mu Chen, Zeyu Zhao, Meng Yang, Roger Frutos, and Jinlong Lv; Methodology: Tian-mu Chen, Zeyu Zhao, Qingqing Hu, Qiuping Chen, Roger Frutos, Zhao Lei, and Mingzhai Wang; Data analysis: Roger Frutos, and Jing-An Cui; Writing - original draft: Tian-mu Chen, Meng Yang, Zeyu Zhao, and Jinlong Lv; Writing - review & editing: Benhua Zhao, Yanhua Su, Yong Chen, and Xu-Sheng Zhang; Data collection: Hao Zhang, Xiongjie Zhai, and Jinlong Lv. All authors have read and agreed to the final version of the manuscript.

## Declaration of competing interest

The authors declare that they have no known competing financial interests or personal relationships that could have appeared to influence the work reported in this paper.
